# Remembering rewarding futures: A simulation‐selection model of the hippocampus

**DOI:** 10.1002/hipo.23023

**Published:** 2018-11-06

**Authors:** Min Whan Jung, Hyunjung Lee, Yeongseok Jeong, Jong Won Lee, Inah Lee

**Affiliations:** ^1^ Center for Synaptic Brain Dysfunctions, Institute for Basic Science Daejeon South Korea; ^2^ Department of Biological Sciences Korea Advanced Institute of Science and Technology Daejeon South Korea; ^3^ Department of Anatomy Kyungpook National University School of Medicine Daegu South Korea; ^4^ Department of Brain and Cognitive Sciences Seoul National University Seoul South Korea

**Keywords:** imagination, memory, place cell, replay, value

## Abstract

Despite tremendous progress, the neural circuit dynamics underlying hippocampal mnemonic processing remain poorly understood. We propose a new model for hippocampal function—the simulation‐selection model—based on recent experimental findings and neuroecological considerations. Under this model, the mammalian hippocampus evolved to simulate and evaluate arbitrary navigation sequences. Specifically, we suggest that CA3 simulates unexperienced navigation sequences in addition to remembering experienced ones, and CA1 selects from among these CA3‐generated sequences, reinforcing those that are likely to maximize reward during offline idling states. High‐value sequences reinforced in CA1 may allow flexible navigation toward a potential rewarding location during subsequent navigation. We argue that the simulation‐selection functions of the hippocampus have evolved in mammals mostly because of the unique navigational needs of land mammals. Our model may account for why the mammalian hippocampus has evolved not only to remember, but also to imagine episodes, and how this might be implemented in its neural circuits.

## 1 INTRODUCTION

The hippocampus is known to play a critical role in encoding certain forms of memory. Despite many years of effort, however, the neural circuit mechanisms underlying the storage and retrieval of memory in the hippocampus remain unclear. Of the numerous theories proposed to explain hippocampal circuit operations so far, the most influential was originally proposed by Marr (1971) and further developed by other investigators (McNaughton & Nadel, 1990; Rolls, & Treves, 1998). In this “standard model”, CA3 stores associative memories based on massive recurrent collateral projections (Amaral, Ishizuka, & Claiborne, 1990) and Hebbian synaptic plasticity (Harris & Cotman, 1986). The dentate gyrus (DG) functions in pattern separation based on expansion recoding (Albus, 1971), which increases the memory storage capacity of CA3. Although this model has been influential for a long time, it is limited in that it does not account for the role of CA1, another major component of the hippocampal tri‐synaptic circuit. Although subsequent studies have proposed numerous functions for CA1 such as match‐mismatch comparison (Hasselmo & Wyble, 1997; Lever et al., 2010; Lisman & Otmakhova, 2001; Vago & Kesner, 2008) and temporal processing (Gilbert, Kesner, & Lee, 2001; Mankin et al., 2012; Rolls & Kesner, 2006), there is no general consensus because of the lack of definitive experimental evidence for one CA1 function over another.

In addition, the standard model, in its current form, is insufficient to explain new experimental findings that suggest a role for the hippocampus in imagining hypothetical episodes (Buckner, 2010; Gaesser, Spreng, McLelland, Addis, & Schacter, 2013; Mullally & Maguire, 2014; Schacter et al., 2012). Amnesic patients with bilateral damage in the medial temporal lobes have trouble imagining future episodes (Andelman, Hoofien, Goldberg, Aizenstein, & Neufeld, 2010; Hassabis, Kumaran, Vann, & Maguire, 2007; Mullally, Intraub, & Maguire, 2012; Race, Keane, & Verfaellie, 2011). Also, as part of the default mode network, the hippocampus is activated not only during autobiographic memory recall, but also while envisioning the future (Addis, Pan, Vu, Laiser, & Schacter, 2009; Addis, Wong, & Schacter, 2007; Botzung, Denkova, & Manning, 2008; Brown et al., 2016; Hassabis, Kumaran, & Maguire, 2007; Okuda et al., 2003; Spreng, Mar, & Kim, 2009; Szpunar, Watson, & McDermott, 2007). In rats, hippocampal place cells go through rapid sequential discharges (replays) that may reflect experienced as well as unexperienced trajectories in association with sharp‐wave ripples [SWRs; large‐amplitude negative potentials (sharp waves) associated with brief high‐frequency oscillations (ripples) (Buzsaki, 2015)] during slow‐wave sleep and awake immobility (Carr, Jadhav, & Frank, 2011; Diba & Buzsaki, 2007; Dragoi & Tonegawa, 2011; Foster & Wilson, 2006; Gupta, van der Meer, Touretzky, & Redish, 2010; Lee & Wilson, 2002; Olafsdottir, Barry, Saleem, Hassabis, & Spiers, 2015; Pfeiffer & Foster, 2013). These results provide converging evidence for the involvement of the hippocampus not only in encoding episodic memories, but also in imagining hypothetical episodes. A new model of the hippocampus, whether it is a revised version of an existing model or an entirely new one, should be able to account for this important aspect of hippocampal function.

In this article, we propose the simulation‐selection model to account for the role of CA1 in memory and imagination. Briefly, the simulation‐selection model posits that CA3 generates both experienced and unexperienced navigation sequences and CA1 selects from among these CA3‐generated sequences, reinforcing those that are likely to maximize reward. The model is speculative at this stage, still requiring significant empirical evidence. Nevertheless, this model is consistent with many of the known features of the hippocampus, provides a new perspective on the role of CA1, and may explain why the mammalian hippocampus has evolved not only to remember, but also to imagine episodes.

## 2 SIMULATION‐SELECTION MODEL

**Figure 1:**
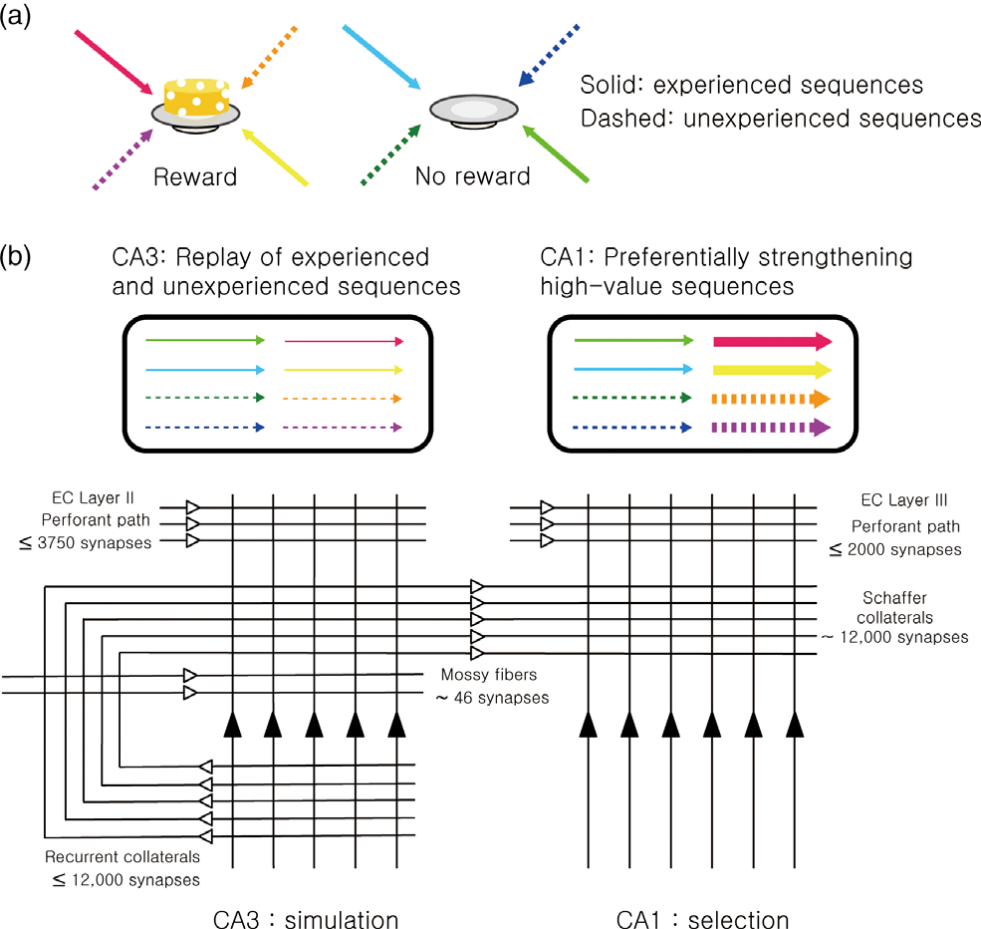
Summary of the simulation‐selection model of the hippocampus. (a) Experienced (solid arrows) and unexperienced (dashed arrows) navigation sequences to two locations where reward was or was not obtained (high‐value and low‐value sequences, respectively) are indicated in different colors. (b) CA3 generates both experienced and unexperienced navigation sequences regardless of value. Of these, CA1 preferentially reinforces high‐value sequences, both experienced and unexperienced. The schematic diagram below shows the basic circuit organizations of CA3 and CA1. The numbers indicate the average number of synapses for each projection pathway for each CA3 and CA1 pyramidal neuron (Amaral et al., 1990). Massive, individually weak recurrent collaterals in CA3 allow the CA3 neural network to generate experienced (remembered) as well as unexperienced (novel) sequences. CA1, which lacks recurrent collateral projections, but conveys strong value signals, preferentially selects and reinforces high‐value sequences [Color figure can be viewed at wileyonlinelibrary.com]

Our model builds on the proposal that the hippocampus generates novel activity patterns for the prediction of future events during offline rest/sleep states (Buckner, 2010; Gershman, Moore, Todd, Norman, & Sederberg, 2012; Gupta et al., 2010; Molter, Sato, & Yamaguchi, 2007; Pezzulo, van der Meer, Lansink, & Pennartz, 2014). Our model concerns only CA3 and CA1, leaving out the first component of the tri‐synaptic circuit, the DG (see Lee & Jung, 2017 for an alternative account of DG function apart from pattern separation). In our model, CA3 neurons generate firing sequences that match previously experienced events (episodic memory recall) as well as novel ones (imagination of hypothetical episodes) based on their recurrent collateral projections. As such, CA3 plays the role of a “simulator”. CA1 neurons preferentially pass on and reinforce high‐value firing sequences based on value‐dependent firing, making CA1 more of a “selector” (Figure 1).

### 2.1 CA3 as a simulator

In our model, both experienced and unexperienced firing sequences are generated in CA3. A neural network with sufficient interconnections among its elements should be able to generate such firing sequences. In particular, a larger number of weak synapses would be more useful than a smaller number of individually strong synapses for generating variable (rather than fixed) sequences. CA3 is the area in the hippocampus most consistent with these features. Specifically, individual neurons in CA3 are connected with one another via a large number of recurrent collateral projections (Amaral et al., 1990) (Figure 1) that are individually weak (Miles & Wong, 1986) even when fully potentiated (Debanne, Gahwiler, & Thompson, 1999). In the rodent hippocampus, place cells often exhibit rapid replays of sequential firing patterns during SWRs, and physiological studies indicate that sharp waves initiated in CA2/CA3 propagate to CA1 (Behrens, van den Boom, de Hoz, Friedman, & Heinemann, 2005; Buzsaki, 1989; Csicsvari, Hirase, Mamiya, & Buzsaki, 2000; Maier, Nimmrich, & Draguhn, 2003; Oliva, Fernandez‐Ruiz, Buzsaki, & Berenyi, 2016; Ylinen et al., 1995). Furthermore, SWR‐associated hippocampal replays may reflect both experienced and unexperienced trajectories (Dragoi & Tonegawa, 2011; Gupta et al., 2010; Olafsdottir et al., 2015) with the direction of a replay proceeding as either a forward or backward version of a recently experienced navigation (Csicsvari, O'Neill, Allen, & Senior, 2007; Diba & Buzsaki, 2007; Foster & Wilson, 2006; Lee & Wilson, 2002; Wikenheiser & Redish, 2013). These results are consistent with a role for CA3 in generating experienced as well as unexperienced firing sequences.

SWR events occurring during offline rest/sleep states provide an opportunity for CA3 to generate spike sequences largely based on internal network dynamics rather than external sensory inputs (Buzsaki, 2015). Replay sequences are perhaps shaped by multiple factors such as anatomical connectivity, current sensory inputs, experience‐dependent synaptic plasticity, recent firing history, and global modulatory signals (e.g., acetylcholine or dopamine) that vary across behavioral states (Atherton, Dupret, & Mellor, 2015; Foster, 2017; Roumis & Frank, 2015). The hippocampus shows strong theta‐frequency oscillation of local field potentials during active exploration of an environment. The spiking of place cells moves earlier in phase relative to the theta oscillation as the animal navigates (theta phase precession; O'Keefe & Recce, 1993; Skaggs, McNaughton, Wilson, & Barnes, 1996). The combination of theta phase precession and spike timing‐dependent plasticity (STDP; Bi & Poo, 1998; Markram, Lubke, Frotscher, & Sakmann, 1997) is thought to facilitate the formation of cell assemblies that tend to fire according to previously experienced discharge sequences (Lengyel, Huhn, & Erdi, 2005; Melamed, Gerstner, Maass, Tsodyks, & Markram, 2004). In conventional STDP, synaptic weight increases with pre‐postsynaptic spike pairing and decreases as the firing sequence reverses (asymmetric STDP). A recent study, however, showed that recurrent collateral synapses in CA3 are potentiated by both forward‐ and reverse‐ordered spike pairing (symmetric STDP) within a relatively broad time window (~150 ms) (Mishra, Kim, Guzman, & Jonas, 2016). If that is the case, during navigation, symmetric STDP would promote profuse associations among CA3 cells with overlapping place fields based on their anatomical connectivity regardless of navigation trajectory. This may facilitate the activation of CA3 cells in diverse sequences during SWRs, including sequences unrelated to experienced navigation trajectories. Together, its massive recurrent collateral projections, individually weak synapses, and symmetric STDP all make CA3 an ideal substrate for the generation of diverse neural activity patterns, both experienced and unexperienced, during offline states.

### 2.2 CA1 as a value‐dependent selector

**Figure 2:**
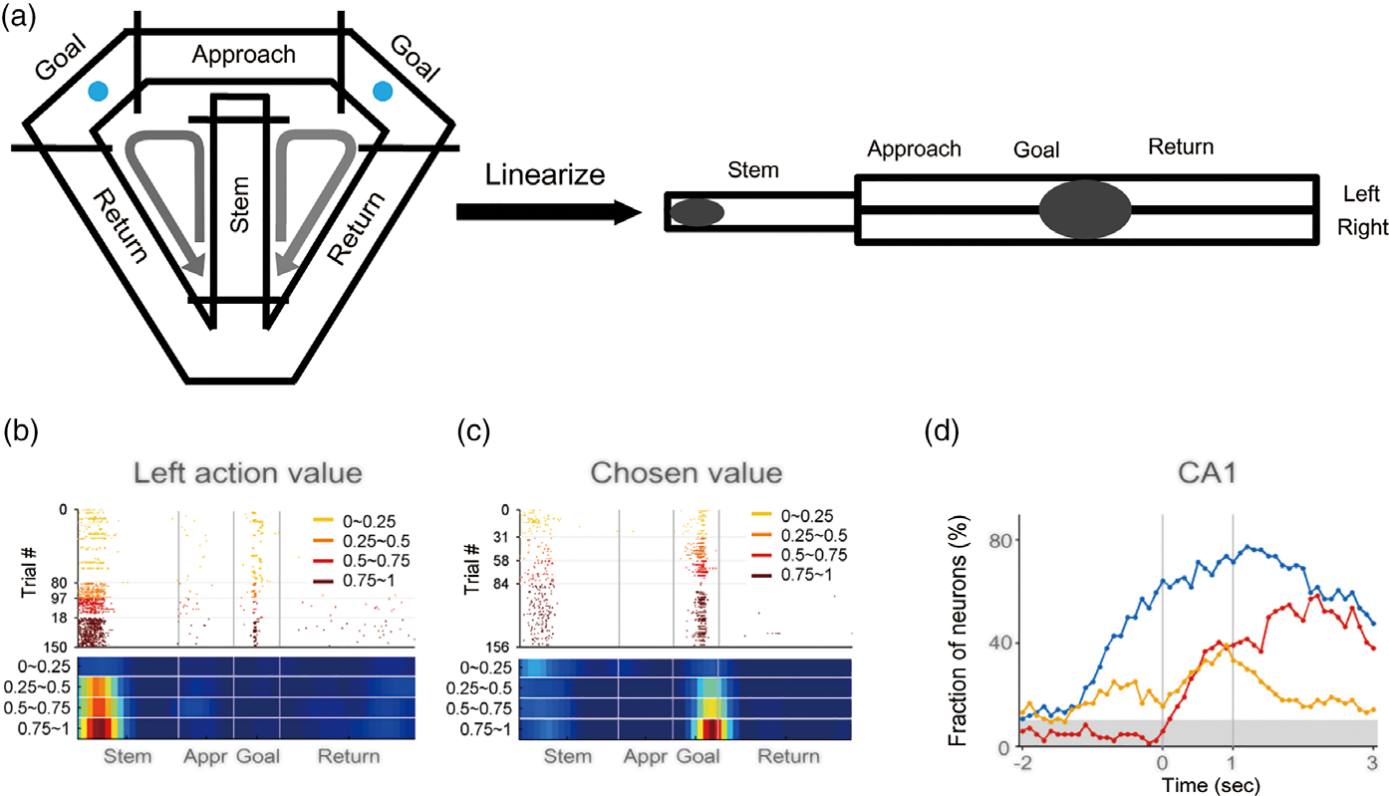
Value‐related neural activity in the rat hippocampus. (a) Modified T‐maze. Water reward was delivered at two goals (blue circles) with different probabilities that changed dynamically across blocks. Spatial firing of hippocampal neurons was analyzed on a linearized maze. (b) an example of an action value‐coding CA1 neuron with its place field on the proximal area of the central stem [indicated by the left ellipse in (a)]. a spatial raster plot (top) and its associated firing rate maps (bottom) are shown. Trials were grouped into quartiles of left action value (0–1; steps of 0.25), computed using a reinforcement learning model, as indicated by the different colors. (c) an example of a chosen value‐coding CA1 neuron with a “merged” place field at the reward site [neural and occupancy data at the left and right reward sites were merged together; indicated by the right ellipse in (a)]. trials were grouped into quartiles of chosen value (i.e., value of the chosen target in a given trial). (d) Convergence of value, reward, and choice signals. Time 0 indicates the time when the trial outcome was revealed at the reward site. The ordinate denotes the fraction of neurons responsive to a given variable (choice, blue; reward, red; chosen value, orange). Shading denotes the chance level. Adapted from Lee et al. (2012) [Color figure can be viewed at wileyonlinelibrary.com]

We propose that CA1 acts as a selective reinforcer, preferentially strengthening high‐value sequences among diverse sequences generated in CA3. Neural activity in CA1 is modulated strongly by both reward (Breese, Hampson, & Deadwyler, 1989; Eichenbaum, Kuperstein, Fagan, & Nagode, 1987; Hollup, Molden, Donnett, Moser, & Moser, 2001; Holscher, Jacob, & Mallot, 2003; Kobayashi, Nishijo, Fukuda, Bures, & Ono, 1997; Smith & Mizumori, 2006; Tabuchi, Mulder, & Wiener, 2003; Watanabe & Niki, 1985; Wirth et al., 2009) and punishment (Berger, Alger, & Thompson, 1976; Berger, Rinaldi, Weisz, & Thompson, 1983; McEchron & Disterhoft, 1997; Moita, Rosis, Zhou, LeDoux, & Blair, 2003; Moita, Rosis, Zhou, LeDoux, & Blair, 2004; Munera, Gruart, Munoz, Fernandez‐Mas, & Delgado‐Garcia, 2001; Segal, Disterhoft, & Olds, 1972). Extending these findings, we discovered that reward value strongly modulates CA1 neural activity in rats performing a dynamic foraging task (Lee, Ghim, Kim, Lee, & Jung, 2012) (Figure 2). Surprisingly, the strength and characteristics of CA1 value signals are indistinguishable from those found in other brain regions implicated in value processing, such as the orbitofrontal cortex and striatum (Kim, Lee, & Jung, 2013; Kim, Sul, Huh, Lee, & Jung, 2009; Sul, Kim, Huh, Lee, & Jung, 2010). This suggests CA1 neurons convey value information just as strongly as other value‐related brain structures do. Furthermore, CA1 value signals show temporal overlap with choice and reward signals as the outcome of the rat's choice is revealed. This indicates all the signals necessary for computing reward prediction error (Sutton & Barto, 1998) and updating reward value converge in CA1 (Figure 2d). Brain imaging studies also found BOLD signals correlated with value (Bornstein & Daw, 2013; Tanaka et al., 2004) in the human hippocampus, although their subregional localization is unclear.

When we compared value‐related neural activity among the subregions of the hippocampus in rats, we found significantly stronger correlates in CA1 than in CA3 (Lee et al., 2017) or the subiculum (Lee et al., 2012). The fact that CA1 conveys stronger value signals than its neighboring input and output structures suggests CA1 plays a more important role in valuation than the other hippocampal subregions. This is consistent with the finding in rats that CA1 neurons, but not CA3 neurons, remap their place fields when rewarding locations are reconfigured (Dupret, O'Neill, Pleydell‐Bouverie, & Csicsvari, 2010). Moreover, chemogenetic inactivation of CA1, but not CA3, CA2 or DG, of the dorsal hippocampus impairs value learning without affecting value‐dependent action selection in mice performing a dynamic foraging task in a modified T‐maze (Jeong et al., 2018). Although additional studies will be required to reveal the details of value information processing in the hippocampus, these results make a strong case for CA1’s role in valuation. Together with the findings that support a role for the hippocampus in imagination (Buckner, 2010; Gaesser et al., 2013; Mullally & Maguire, 2014; Schacter et al., 2012), these findings imply that the contents of remembered and imagined episodes are modulated by value in CA1. We argue that this feature distinguishes CA1 from the other subregions in the hippocampus.

In support of our proposal, ample evidence suggests CA1 replays are biased toward reward locations. Although the frequency of SWR‐associated CA3 reactivation is enhanced by reward, there is no preferential reactivation of CA3 neurons with place fields near reward locations (Singer & Frank, 2009). In contrast, CA1 neurons with place fields near reward locations show a stronger tendency to fire together during SWRs than those with place fields farther from reward locations (Dupret et al., 2010). Furthermore, trajectories reconstructed from replays of CA1 place cells are preferentially directed toward previously visited as well as unvisited (but observed) reward locations (Foster & Wilson, 2006; Gupta et al., 2010; Olafsdottir et al., 2015; Pfeiffer & Foster, 2013; Singer & Frank, 2009). This may relate to enhanced imagination of episodic future events by reward in humans (Bulganin & Wittmann, 2015). Hippocampal activity patterns for high‐reward contexts are also preferentially reactivated during post‐learning rest, and this reactivation is predictive of memory retention in humans (Gruber, Ritchey, Wang, Doss, & Ranganath, 2016).

Based on the abovementioned findings, we propose here that value‐dependent replays in CA1 help an animal to navigate along an optimal route from an arbitrary starting location. Specifically, frequently replayed rewarding sequences in CA1, both experienced and unexperienced, are strengthened during SWRs so that they are preferentially activated during subsequent navigation. We propose two different mechanisms for strengthening rewarding sequences during SWRs. First, frequently replayed CA1 sequences may be stored in downstream brain structures, such as the prefrontal cortex (Frankland & Bontempi, 2005; Jung, Baeg, Kim, Kim, & Kim, 2008), over time. An animal may be able to choose an optimal route to a target destination based on an array of rewarding sequences stored in downstream brain structures, such that hippocampal lesions do not impair behavioral performance in tests that require recall of remote memories of rewarding trajectories. According to this scenario, memory consolidation is a process of actively selecting and reinforcing high‐reward action (or event) sequences rather than passively storing incidental events. Second, frequently replayed CA1 sequences during SWRs may be strengthened within the hippocampus (Buzsaki, 2015; Csicsvari & Dupret, 2014). Studies show that hippocampal place cell activity during theta‐frequency state (such as during locomotion) is related to both current and future positions (Ainge, Tamosiunaite, Woergoetter, & Dudchenko, 2007; Gupta, van der Meer, Touretzky, & Redish, 2012; Johnson & Redish, 2007; Pastalkova, Itskov, Amarasingham, & Buzsaki, 2008; Wikenheiser & Redish, 2015; Zheng, Bieri, Hsiao, & Colgin, 2016; see also Lisman & Redish, 2009; Mehta, 2001; Samsonovich & Ascoli, 2005; Sanders, Renno‐Costa, Idiart, & Lisman, 2015; Stachenfeld, Botvinick, & Gershman, 2017). Hippocampal neural plasticity during SWRs may bias such “planning”‐related, theta‐state hippocampal neural activity toward rewarding locations from an arbitrary position. These two mechanisms may work in parallel, but perhaps with different time courses, to allow an animal to choose an optimal route from an arbitrary starting location.

## 3 IMPLEMENTATION OF THE SIMULATION‐SELECTION MODEL

**Figure 3:**
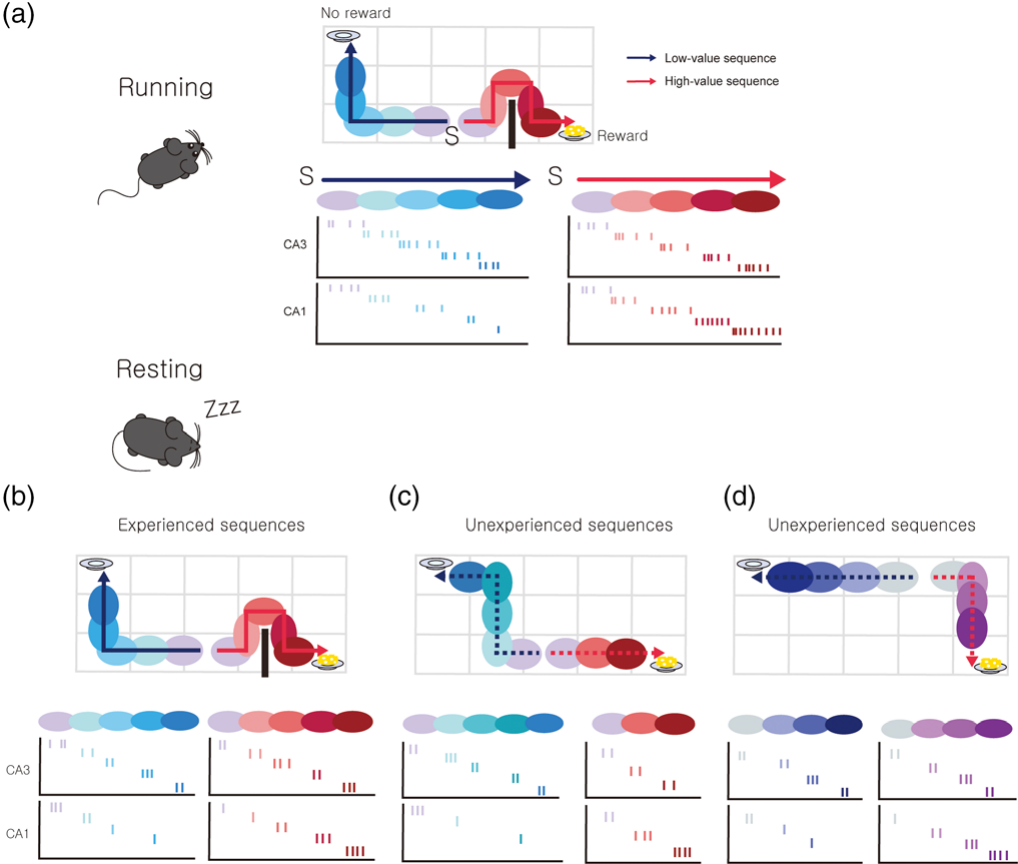
A potential neural mechanism for CA1 to preferentially replay high‐value navigation sequences. (a) Hypothetical value‐dependent discharges of CA3/CA1 neurons during locomotion. Top, an animal is assumed to have visited all locations, but experienced only two specific navigation sequences between a particular starting location (S) and two target locations, one of which led to a reward (bottom right corner). Each ellipse represents a place field for a CA3 or CA1 neuron. The thick black vertical line indicates a physical barrier. Bottom, each set of spikes (tick marks) represents hypothetical place cell discharges for the navigation sequences shown on top. Assuming that CA1 neurons increase firing with increasing value, CA1 neuronal firing rates would be low during travel to the no‐reward site (low‐value sequence) so that the chance for CA3 and CA1 neurons with overlapping place fields to fire together is low (left). In contrast, CA1 neuronal firing rates would be high during travel to the reward site (high‐value sequence) so that the chance for CA3 and CA1 neurons with overlapping place fields to fire together is high (right). As a result, synaptic connections between CA3 and CA1 neurons with overlapping place fields are more likely to be strengthened by activity‐dependent plasticity for the high‐value sequence. (b–d) hypothetical replay sequences during SWRs. Top, solid and dashed arrows indicate experienced and unexperienced navigation sequences, respectively. Bottom, each set of spikes represents hypothetical SWR‐associated replays of CA3 and CA1 place cells for the corresponding navigation sequences shown on top. (b) Previously experienced navigation sequences. (c) Unexperienced navigation sequences starting from the same initial location (S) shown in (a). (d) Unexperienced navigation sequences starting from a new location. When CA3 generates an experienced or unexperienced sequence leading to the no‐reward site, the chance for the same sequence to be replayed in CA1 is low because of the weak synaptic weights between the CA3 and CA1 neurons. When CA3 generates an experienced or unexperienced sequence that leads to a reward, however, the chance for the same sequence to be replayed in CA1 is high because of the strong synaptic weights between the CA3 and CA1 neurons. The hypothetical spike firing in (c, d) illustrates how unexperienced, yet high‐value navigation sequences can be replayed during SWRs. In a future incident, if the barrier is removed, the animal is likely to take the path shown in (c) instead of that in (b) as a short cut from the original starting point (S) [Color figure can be viewed at wileyonlinelibrary.com]

At this stage, we can only speculate regarding *how* CA1 selects high‐value sequences because relatively little is known about the value‐related discharge characteristics of hippocampal neurons. Nevertheless, we propose value‐dependent changes in CA3‐CA1 connection strengths are responsible for CA1’s role in sequence selection. Let's assume CA1 neurons show value‐dependent firing such that the closer the place field gets to a reward site, the higher its firing rate (Figure 3a). This is consistent with the findings that CA1 neurons increase firing near reward locations (Breese et al., 1989; Dupret et al., 2010; Hollup et al., 2001; Kobayashi, Tran, Nishijo, Ono, & Matsumoto, 2003; Mamad et al., 2017). Let's imagine that an animal has explored its environment sufficiently so that all areas of the environment were covered. CA3 and CA1 neurons with overlapping place fields will fire together during exploration, and, assuming dependence of CA1 place cell activity on the distance to a reward site, coincident firing would occur more often in CA1 neurons with place fields closer to a reward site (Figure 3a). Then, after sufficient exploration, the synapses between those CA3 and CA1 neurons with overlapping place fields near a reward (or non‐reward) location should be strongly (or weakly) potentiated via activity‐dependent synaptic plasticity. Even though precise rules and factors governing CA3–CA1 STDP are not fully understood (Edelmann, Cepeda‐Prado, & Lessmann, 2017; Kwag & Paulsen, 2009; Nishiyama, Togashi, Aihara, & Hong, 2010; Sugisaki, Fukushima, Tsukada, & Aihara, 2011; Tsukada, Aihara, Kobayashi, & Shimazaki, 2005; Wittenberg & Wang, 2006), CA3/CA1 place cell spike patterns recorded in freely moving rats induced long‐term potentiation at CA3–CA1 synapses, demonstrating naturally occurring spike patterns during navigation can potentiate CA3–CA1 synapses (Isaac, Buchanan, Muller, & Mellor, 2009). Such activity‐dependent synaptic weight changes may lead to the preferential replaying of rewarding sequences, both experienced and unexperienced, in CA1 during subsequent offline SWR episodes (Figure 3b–d). This model is consistent with the finding that neuronal coactivity between CA3 and CA1 during exploration is correlated with that during SWRs in a subsequent sleep period (O'Neill, Senior, Allen, Huxter, & Csicsvari, 2008). Modeling studies also suggest the strength of the CA3 excitatory inputs to CA1 pyramidal cells strongly influence the spiking activity of CA1 pyramidal cells during SWRs (Taxidis, Coombes, Mason, & Owen, 2012; Taxidis, Mizuseki, Mason, & Owen, 2013). In this way, CA1 acts as a filter, facilitating the replay of CA3‐generated sequences that lead to a reward.

It is relatively straightforward to imagine that frequently replayed CA1 rewarding sequences are stored permanently in downstream brain structures over time (Buzsaki, 1996; Csicsvari & Dupret, 2014; Diekelmann & Born, 2010; Frankland & Bontempi, 2005; Girardeau & Zugaro, 2011; O'Neill, Pleydell‐Bouverie, Dupret, & Csicsvari, 2010). There are some issues to consider, however, regarding how frequently replayed CA1 sequences are strengthened within the hippocampal network to help flexible navigation. One issue is related to synaptic plasticity during SWRs. It is currently controversial whether hippocampal synapses are potentiated or exclusively depressed during SWRs (Bukalo, Campanac, Hoffman, & Fields, 2013; Buzsaki, 2015; Buzsaki, Haas, & Anderson, 1987; Colgin, Kubota, Jia, Rex, & Lynch, 2004; King, Henze, Leinekugel, & Buzsaki, 1999; Leonard, Mcnaughton, & Barnes, 1987; Norimoto et al., 2018; Sadowski, Jones, & Mellor, 2016; Tononi & Cirelli, 2014). Nevertheless, both synaptic potentiation and depression have been proposed as mechanisms for memory consolidation (Bukalo et al., 2013; Norimoto et al., 2018; Sadowski et al., 2016; Tononi & Cirelli, 2014). Those hippocampal synapses supporting frequently replayed rewarding sequences may be further strengthened and/or other synapses are weakened during SWRs, so that relative strengths of frequently replayed sequences are enhanced. Another issue is related to generating sequences for novel rewarding trajectories during theta‐frequency state. Value‐dependent CA3–CA1 synaptic weight changes during exploration (Figure 3a) would be sufficient to preferentially activate CA1 sequences for *experienced* rewarding trajectories during theta‐frequency state. However, it may be insufficient to drive CA1 sequences for *novel* rewarding trajectories because the degree of freedom for CA3‐generated sequences is expected to be low during theta‐frequency state, during which external sensory influence and inhibitory tone are relatively strong (Buzsaki, 1989). We propose hippocampal synaptic plasticity during SWRs, during which diverse CA3 sequences are generated, may alleviate this constraint. Redistribution of CA3–CA3 synaptic weights and strengthening diverse combinations of CA3–CA1 cell pairs during SWRs may increase the diversity of planning‐related CA1 neural activity during subsequent navigation. These processes, in combination with value‐dependent firing of CA1 neurons, may allow planning‐related CA1 neural activity during subsequent navigation to include those representing novel rewarding trajectories. Again, empirical evidence is largely lacking for the proposed neural processes. Much investigation is needed to understand how value‐dependent CA1 replays might be translated into flexible navigation.

**Figure 4:**
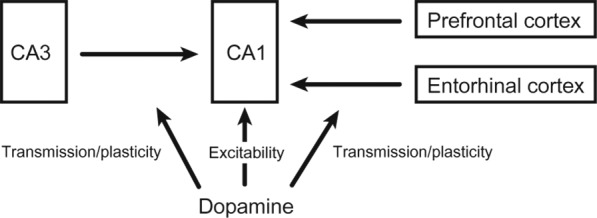
A schematic showing inputs to CA1 that may control the value‐dependent activity of CA1 neurons

**Figure 5:**
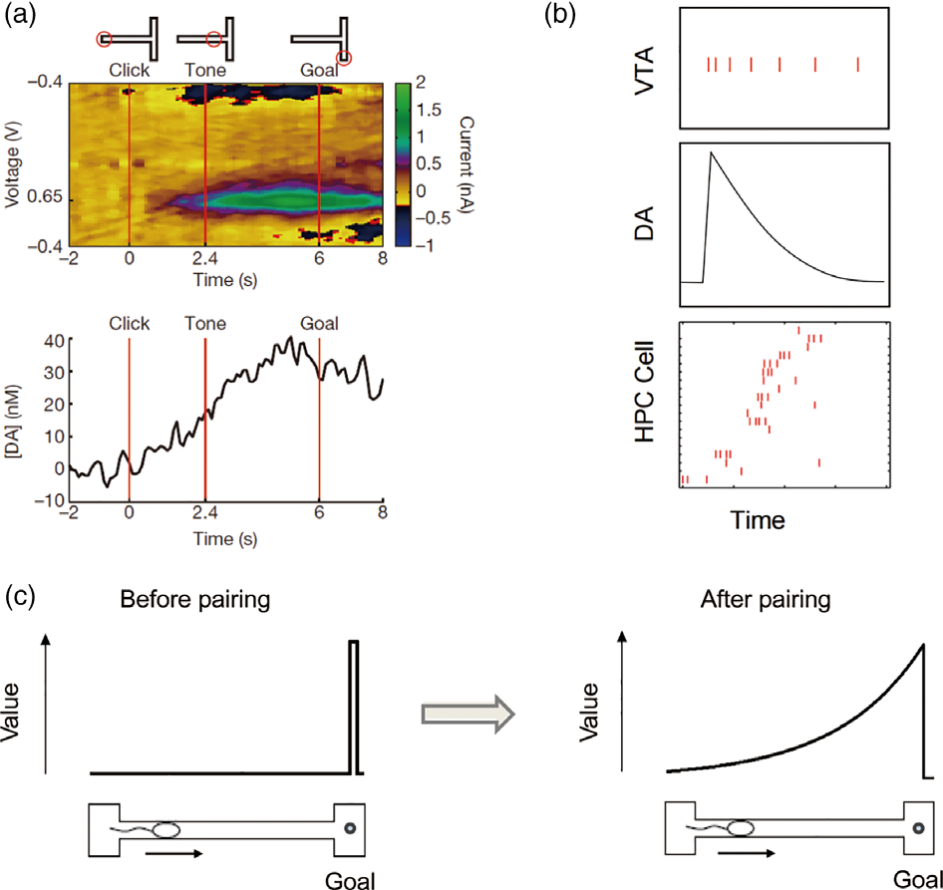
Hypothetical role of dopamine in modulating the value‐dependent firing of CA1 neurons. (a) Dopamine concentration in the striatum during maze learning. Adapted with permission from Howe et al. (2013). (b, c) reverse replay of CA1 place cells and a model in which dopamine associates value to each place cell according to its distance from a reward site. HPC, hippocampus; DA, dopamine; VTA, ventral tegmental area. Adapted with permission from Foster and Wilson (2006) [Color figure can be viewed at wileyonlinelibrary.com]

How do CA1 neurons fire in a value‐dependent manner? Value‐related activity of CA1 neurons may be controlled independent of CA3–CA1 projections. It may be driven by monosynaptic inputs from extrahippocampal structures such as the prefrontal cortex (Rajasethupathy et al., 2015), which conveys value signals (Barraclough, Conroy, & Lee, 2004; Kim, Hwang, & Lee, 2008; Sul et al., 2010), or the entorhinal cortex (Witter, Doan, Jacobsen, Nilssen, & Ohara, 2017; Figure 4). Neuromodulatory inputs, such as dopamine, may also contribute to value‐related activity of CA1 neurons. Howe, Tierney, Sandberg, Phillips, and Graybiel (2013) showed dopamine concentration in the striatum of rats running a T‐maze gradually increases between the starting and goal locations (Figure 5a). Assuming a similar dopamine concentration gradient in CA1, dopamine may control the excitability of CA1 neurons such that their activity increases as an animal nears a reward location. Alternatively, dopamine may contribute to the value‐dependent firing of CA1 neurons by modulating the transmission and/or plasticity of projections from external structures such as entorhinal or prefrontal cortical projections (Ito & Schuman, 2007; Figure 4).

An alternative possibility is that the CA3 projections to CA1 mediate both its value‐related activity and subsequent role in filtering. For example, dopamine may control synaptic transmission (Rosen, Cheung, & Siegelbaum, 2015) and/or plasticity (Edelmann et al., 2017; Frey & Morris, 1998; Frey, Schroeder, & Matthies, 1990; Li, Cullen, Anwyl, & Rowan, 2003; Navakkode, Sajikumar, & Frey, 2007; O'Carroll & Morris, 2004; Otmakhova & Lisman, 1996) in CA3–CA1 connections in a value‐dependent manner (Figure 4). The putative dopamine concentration gradient during exploration (Howe et al., 2013) may control the transmission/plasticity of CA3–CA1 connections during exploration such that CA1 neurons show value‐dependent neural activity during exploration and also mediate preferential replays of high‐value sequences during an offline state (Figure 3). It is also possible that the combination of reverse replays and phasic dopaminergic activity, which conveys reward prediction error signals (Cohen, Haesler, Vong, Lowell, & Uchida, 2012; Roesch, Calu, & Schoenbaum, 2007; Schultz, Dayan, & Montague, 1997), may act to assign value to CA1 place cells. CA1 place cells are often replayed in the reverse order when an animal arrives at a goal location (Ambrose, Pfeiffer, & Foster, 2016; Foster & Wilson, 2006). Assuming that dopamine signals in CA1 peak at the time of arrival at a goal and then decay gradually over time, those cells with their place fields closer to the goal may be more strongly paired with dopamine signals (Foster & Wilson, 2006) (Figure 5b,c). This would also assign value to CA1 place cells according to the distance between a place field and a reward site, and it could be accomplished by several different mechanisms including the modification of synaptic weights in the CA3–CA1 connections (Figure 4). The existing evidence for this hypothesis, although limited, is consistent with a role for dopamine in the value‐related activity of CA1 neurons. Inactivation of the ventral tegmental area (VTA) affects the spatial firing of CA1, but not CA3, place cells in rats (Martig & Mizumori, 2011). Hippocampal place cell stability is enhanced (or reduced) by a D1/D5 receptor agonist (or antagonist) in mice (Kentros, Agnihotri, Streater, Hawkins, & Kandel, 2004), and CA1 place cell activity is weak and unstable in D2 receptor‐knockout mice (Nguyen et al., 2014). In addition, optogenetic stimulation of VTA dopaminergic neurons increases the firing rates of CA1 place cells and shifts place fields toward locations associated with VTA stimulation in rats (Mamad et al., 2017). There is also evidence linking dopaminergic activity during exploration to replays during a subsequent rest period. Optogenetic stimulation of dopaminergic fibers in mice exploring a novel environment enhances CA1 reactivation of waking firing patterns during a subsequent sleep/rest session (McNamara, Tejero‐Cantero, Trouche, Campo‐Urriza, & Dupret, 2014). Here we consider dopamine as a potential mediator for CA1 value signals. However, other neuromodulatory systems, such as septal cholinergic inputs (Everitt & Robbins, 1997), may well be involved in mediating CA1 value signals, which remains to be explored.

Although it is unknown how CA1 neurons acquire value‐dependent firing, studies indicate that spatial firing of CA1 neurons can be changed relatively easily compared to that of CA3 neurons. As mentioned above, spatial firing in CA1, but not in CA3, is altered by reward (Dupret et al., 2010) or VTA inactivation (Martig & Mizumori, 2011). Spatial firing in CA1 changes gradually over time, whereas spatial firing in CA3 is maintained stably over time (Mankin et al., 2012; Manns, Zilli, Ong, Hasselmo, & Eichenbaum, 2007). Neural activity in CA1, but not in CA3, is enhanced in a novel environment (Karlsson & Frank, 2008). Furthermore, changes in CA1 spatial firing can be induced rapidly. CA1 place cells, once suppressed, become unstable and remap their firing fields (Schoenenberger, O'Neill, & Csicsvari, 2016). Also, even a single episode of intracellular stimulation of a CA1 neuron, if sufficiently strong, may produce a new place field at the stimulation location (Bittner et al., 2015). By contrast, activation/inhibition of CA3 place cells by optogenetic mossy fiber stimulation induces only transient changes in CA3 spatial firing (Lee et al., 2018). These results suggest that CA1 spatial firing may change flexibly according to changes in the environment, especially those related to reward, so that it can guide the animal's navigation in an adaptive manner.

We certainly do not intend to argue that value‐dependent strengthening of CA3–CA1 synapses during exploration is the only way for value‐dependent CA1 neural activity to influence the content of replays. Studies suggest persistent reinforcement signals by dopaminergic neurons influence hippocampal neural activity during post‐learning rest. In rodents, VTA neuronal activity during task performance is replayed in association with hippocampal SWRs during a subsequent rest period (Valdes, McNaughton, & Fellous, 2015). In addition, the pairing of the spikes of a particular place cell with medial forebrain bundle stimulation during sleep induces a preference for the location encoded by that place cell, such that the animal spends more time in the location encoded by the stimulated place cell (de Lavilleon, Lacroix, Rondi‐Reig, & Benchenane, 2015). In humans, a dopamine agonist given during sleep enhances memories of low‐reward stimuli to the level of high‐reward stimuli (Feld, Besedovsky, Kaida, Munte, & Born, 2014). The midbrain dopaminergic system and the hippocampus also show interactions during post‐learning rest, and these interactions are correlated with the retention of objects learned in high‐reward contexts (Gruber et al., 2016). These studies suggest that the hippocampus may work with other related neural systems in controlling value‐dependent CA1 replays.

So far, for the sake of simplifying our argument, we have considered only CA1 neurons whose activity increases with value. However, CA1 also contains neurons whose activity decreases as a function of value (Lee et al., 2012) and there exists extensive literature regarding the role of the hippocampus in contextual fear conditioning (LeDoux, 2000; Maren, Phan, & Liberzon, 2013) and avoidance behavior (Cimadevilla, Wesierska, Fenton, & Bures, 2001; Kubík, Stuchlik, & Fenton, 2006; Lorenzini, Baldi, Bucherelli, Sacchetti, & Tassoni, 1996; Olton & Isaacson, 1968; Telensky et al., 2011). CA1 neurons whose firing decreases as a function of value may respond particularly strongly to aversive stimuli (Berger et al., 1976; Berger et al., 1983; McEchron & Disterhoft, 1997; Moita et al., 2003; Moita et al., 2004; Munera et al., 2001; Segal et al., 1972), serving to ensure recall of places/trajectories to avoid (Wu, Haggerty, Kemere, & Ji, 2017). In other words, distinct CA1 populations may represent trajectories to both choose and avoid. It will be interesting to examine whether CA1 neurons encoding positive and negative values have different output connectivity. The former may preferentially activate reward‐related areas such as the ventromedial prefrontal cortex and ventral striatum (c.f., Kuhl, Shah, DuBrow, & Wagner, 2010), while the latter may activate punishment‐related areas such as the amygdala (c.f., Ciocchi, Passecker, Malagon‐Vina, Mikus, & Klausberger, 2015). Alternatively, CA1 neurons may indiscriminately elevate activity in response to both appetitive and aversive events, so that CA1 place cell sequences representing trajectories toward both reward and punishment locations are preferentially replayed during SWRs. If so, decisions to or not to approach a location must take place in a brain structure downstream of the hippocampus. It is also possible that, like midbrain dopaminergic neurons (Bromberg‐Martin, Matsumoto, & Hikosaka, 2010), some CA1 neurons elevate activity in response to both reward and punishment, while others increase activity exclusively to reward. Distinct patterns of CA1 neuronal population activity, along with synaptic plasticity, may instruct downstream motor‐related structures to approach or move away from a certain location. Currently, value‐dependent hippocampal neuronal activity, especially value‐dependent replays, are poorly understood. It will be important in the future to characterize value‐dependent CA1 neuronal activity under different behavioral states. It will be also important to compare value‐dependent replays of CA1 and CA3 neurons, as our model predicts that CA3 replays are less dependent on value than CA1 replays.

## 4 WHY SIMULATION‐SELECTION? A NEUROECOLOGICAL PERSPECTIVE

**Figure 6:**
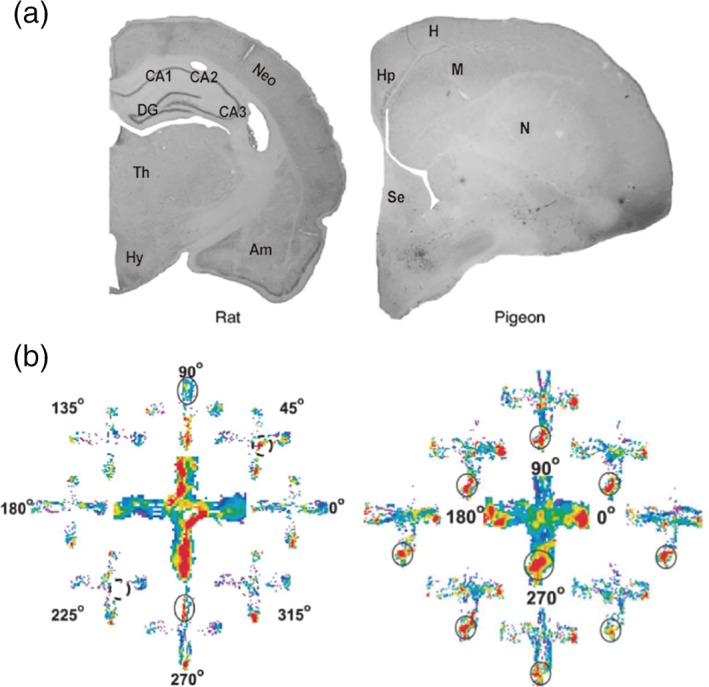
The structure and physiology of the avian hippocampus differ from those of the mammalian hippocampus. (a) Coronal sectional views of rat and pigeon brains. Adapted with permission from Bingman, Salas, & Rodriguez, 2009. Rat, DG, dentate gyrus; neo, neocortex; Hy, hypothalamus; am, amygdala; pigeon, Hp, hippocampus; H, hyperpallium; M, mesopallium; N, nidopallium; se, septum. (b) Examples of spatial firing of hippocampal neurons in pigeons. Left, a “path firing” neuron recorded from the left hippocampus. Right, a “location firing” neuron recorded from the right hippocampus. Note the elevated activity (red color) near all goals (the ends of each arm) for the location firing neuron. Adapted with permission from Siegel, Nitz, and Bingman (2006) [Color figure can be viewed at wileyonlinelibrary.com]

We propose that the simulation‐selection process in the hippocampus might reflect the unique ecological needs of navigating mammals. The hippocampus supports allocentric spatial memory in many different animal species (Herold, Coppola, & Bingman, 2015; Striedter, 2016). Among them, warm‐blooded animals (i.e., mammals and birds) show far better spatial memory capacity than cold‐blooded animals. For example, some food‐caching birds can store foods at thousands of locations and retrieve them successfully over a period of many months (Cowie, Krebs, & Sherry, 1981; Shettleworth, 1990; Stevens & Krebs, 1986). The anatomical structure of the hippocampus in birds, however, is quite different from the well‐conserved structure observed in mammalian species (Figure 6a) (Manns & Eichenbaum, 2006). The avian hippocampus, for example, does not have well‐differentiated cell layers like the highly differentiated layers of the mammalian hippocampus. As a result, in the avian hippocampus, it is difficult to identify the subfields (e.g., DG, CA3, and CA1) that are so distinct in the mammalian hippocampus. Whereas information flow tends to be unidirectional in the mammalian hippocampus, connections between the major hippocampal divisions in birds are mostly reciprocal (Herold et al., 2015; Striedter, 2016). There is also no direct projection from the medial septum to the hippocampus in birds like there is in mammals (Krayniak & Siegel, 1978). Thus, avian and mammalian hippocampi seem to have been evolving separately for quite some time (Herold et al., 2015; Striedter, 2016). Although it is possible that mammals and birds came up with different solutions for the problem of allocentric spatial memory, their divergent hippocampal structures suggest avian and mammalian hippocampi have evolved to cope with different evolutionary needs.

We propose that the mammalian hippocampus has evolved into its current form to meet the special ecological demands of ground‐based navigation. Birds fly and mammals run. Birds may not need to concern much about specific routes between their current position and their destination under most circumstances because they can travel directly “as the crow flies” to their destination. Mammals, in contrast, must remember not only their destination but also the various routes by which they can get there. This is because obstacles (i.e., trees, rivers, pits, rocks, etc.) often stand between their current position and their destination. Mammals, therefore, benefit from the ability to come up with novel, efficient routes to their destinations from arbitrary starting places (Gallistel, 1990; Tolman, 1948). Imagine a vole searching for food in its territory. If a predator appears, the vole must run back to its home burrow via the shortest available route from its current location. This requires the ability to choose an optimal route to a goal from an arbitrary starting location. Therefore, whereas birds seem to need to know only the location of their final destination to reach it via a beeline path in the sky, land mammals have more complex navigational demands that require them to prepare for efficient navigation between arbitrary locations. Evidence suggests that the avian hippocampus, unlike the mammalian hippocampus, may be dedicated to processing spatial information (Herold et al., 2015). The need to process and remember objects and events associated with route‐dependent navigation may have facilitated the extension of the functions of the mammalian hippocampus from spatial memory and planning to more general episodic memory and planning, especially in higher animals (Buzsaki & Moser, 2013).

Although there exist only a small number of physiological studies in birds, they are consistent with our proposal. In pigeons, hippocampal neurons do not show the same discharge characteristics as mammalian place cells. Although recorded during walking rather than flying, cells in the right hippocampus fire preferentially at goals (“location firing”) while those in the left hippocampus tend to fire in association with paths connecting goals (“path firing”; Figure 6b) (Siegel et al., 2006; Siegel, Nitz, & Bingman, 2005). This “spatial” firing is diminished greatly when pigeons forage for randomly scattered food (unstable goal locations) (Kahn, Siegel, Jechura, & Bingman, 2008), suggesting that the pigeon hippocampus is primarily concerned with goal locations and the direct paths between them. In the mammalian hippocampus, in contrast, place cells show spatial firing patterns that cover an entire environment regardless of the presence of a stable goal location (Muller, Kubie, & Ranck, 1987). This allows mammals to construct arbitrary routes by connecting place cells. Another physiological difference is the absence of SWRs in birds. So far, no one has observed SWRs in the avian hippocampus (Rattenborg, Martinez‐Gonzalez, Roth, & Pravosudov, 2011). Although uncertainty remains due to the small number of studies on the avian hippocampus, the avian hippocampus seems to be primarily concerned with goal locations and the direct paths between them, whereas the mammalian hippocampus seems to have evolved to select optimal routes between arbitrary locations. How do mammals accomplish this? Because it would be difficult and time‐consuming to remember all possible routes after physically experiencing each one, mammals may accomplish this by simulating diverse hypothetical navigation sequences using spatial information collected during actual navigations and then selecting high‐value sequences. We propose that the mammalian hippocampus evolved for this purpose, to generate arbitrary navigation sequences and select those of high value.

On a related note, it would be of interest to examine the hippocampus of mammals such as bats and cetaceans that abandoned ground navigation. Bats have well developed hippocampi (Manns & Eichenbaum, 2006) and physiological studies have found various spatial cells including “place” cells with three‐dimensional firing fields in the hippocampus (Geva‐Sagiv, Las, Yovel, & Ulanovsky, 2015). In contrast, cetaceans have very small hippocampi relative to the total brain size (Patzke et al., 2015). Does this difference reflect different navigation demands between bats and cetaceans? Cetaceans spend most of time in the ocean, thus the demand for remembering specific navigation routes may be low. Bats are less efficient in flight, but more efficient in maneuvering than birds (Hedenstrom & Johansson, 2015). They typically spend a large amount of time in enclosed spaces such as caves, although sometimes fly a long distance like birds (Geva‐Sagiv et al., 2015), so that the demand for remembering diverse navigation routes may be still high. The amount of studies on bat and cetacean navigation is small compared to that on rodent navigation. Additional studies on this issue may provide valuable insights regarding our hypothesis.

## 5 SIMULATION DURING RESTING STATE

We have argued that the mammalian hippocampus may have evolved to solve the problem of finding optimal routes between two arbitrary locations and that it accomplishes this by simulating diverse navigation sequences and selecting from among them. It may seem simpler and perhaps more economical to calculate optimal routes on an as‐needed basis using the spatial information represented in the hippocampus instead of going through the process of simulation and selection in advance. In fact, there is evidence suggesting that the rat hippocampus actually does this. As mentioned above, hippocampal place cell activity during navigation is related to both current and anticipated future positions (Gupta et al., 2012; Wikenheiser & Redish, 2015; Zheng et al., 2016). In addition, rats often show vicarious trial‐and‐error behavior (i.e., horizontal head movements alternating between potential choices at a choice point) during an early stage of learning (Redish, 2016). During such behaviors, spatial representations reconstructed from CA3 ensemble activity often transiently shift ahead of the animal's current position, suggesting that the animal is “thinking ahead” about possible future navigation routes (Johnson & Redish, 2007). Furthermore, CA1 place cells in rats show relatively slow sequential discharges before navigation onset—firing in the range of seconds instead of milliseconds—that predict the animal's future behavioral choices (Ainge et al., 2007; MacDonald, Carrow, Place, & Eichenbaum, 2013; Pastalkova et al., 2008). These types of neural activity are observed in association with theta oscillations rather than SWRs, which is consistent with the finding that pharmacological disruption of the theta dynamics of rat hippocampal place cells impairs performance in a delayed alternation task (Robbe et al., 2006; Robbe & Buzsaki, 2009). Evidence suggests that planning of future navigation also happens during SWRs. The direction of SWR‐associated hippocampal replays during brief waking immobility (“exploratory” SWRs; Atherton et al., 2015) is correlated with the direction of the animal's future navigation (Diba & Buzsaki, 2007; Pfeiffer & Foster, 2013; Wu et al., 2017). Also, spatial working memory performance is correlated with coordinated CA3/CA1 neuronal activity during awake SWRs (Singer, Carr, Karlsson, & Frank, 2013) and impaired by blockade of awake SWRs (Jadhav, Kemere, German, & Frank, 2012). These results suggest the rat hippocampus, while being actively engaged in behavior, is able to process information related not only to the current spatial position, but also to trajectories to potential targets.

Why, then, does the hippocampus adopt such a seemingly inefficient and energy‐consuming solution of simulating diverse navigation trajectories from arbitrary locations? We suggest that this strategy provides a survival advantage. Advanced simulation‐selection abilities would prepare an animal for identifying optimal navigational routes between arbitrary locations. Otherwise, the animal would be forced to come up with the optimal solution only when it becomes necessary, a process that may take too much time. In an emergency situation, such as when being chased by a predator, this could be disastrous. Although it may be energy‐intensive, if we assume that advanced simulation‐selection of potential navigation routes improves survival in mammals, it would be least wasteful to perform this operation during rest or sleep states when an animal is not engaged in the active processing of external information (Buckner, 2010). We suggest that hippocampal replays observed during sleep and rest states represent the process of simulating hypothetical navigation routes while the brain is in idler states. Brain imaging studies have reported rat brain activity patterns that resemble the default mode network activity found in humans (Gozzi & Schwarz, 2016; Lu et al., 2012). As the hippocampus is considered an older cortex in the brain, its evolution into a neural substrate for simulation and selection during idle states may have contributed to the evolution of the default mode network.

## 6 COMPARISON WITH OTHER THEORIES

The unique feature of our model is that the core function of CA1 (i.e., the selection of high‐value sequences) relies on value‐dependent discharges of CA1 neurons. Traditionally, the hippocampus has been considered a computational area where cognitive variables related to spatial navigation and episodic memory are computed and represented. Although memories of different places and events are associated with certain emotions and valences, emotions and valences are largely assumed to be represented elsewhere rather than in the hippocampus itself. In previous models of the hippocampus, CA1 has been proposed to serve such functions as match‐mismatch comparison or novelty detection (Hasselmo & McClelland, 1999; Hasselmo & Wyble, 1997; Lever et al., 2010; Levy, 1989; Lisman & Otmakhova, 2001; Vago & Kesner, 2008), pattern completion (Cheng, 2013; Rolls, 2016), processing temporal information (Gilbert et al., 2001; Mankin et al., 2012; Rolls & Kesner, 2006), transforming CA3 representations for neocortical projections (Kesner & Rolls, 2015; McClelland & Goddard, 1996), and behavioral inhibition (Bannerman et al., 2012). These functions are related to spatial/cognitive processes, but not valuation. As mentioned earlier, recent studies suggest that CA1 may be uniquely involved in valuation among different hippocampal subregions (Jeong et al., 2018; Lee et al., 2012; Lee et al., 2017). Our model assumes that CA1 value processing is related to its core function (selecting high‐value sequences), whereas most existing models, to the best of our knowledge, do not consider valuation itself as one of the major underlying variables that affect CA1 functions. In this respect, our model is distinct from existing models of the hippocampus.

Previous theoretical attempts to link planning‐related hippocampal activity to reward/value‐dependent learning assumed that sequences generated in the hippocampus are evaluated in other value‐processing brain structures such as the ventral striatum (Gershman et al., 2012; Johnson, van der Meer, & Redish, 2007; Pezzulo et al., 2014; van der Meer, Kurth‐Nelson, & Redish, 2012; Yu & Frank, 2015). In contrast, our model suggests value‐related discharges of CA1 neurons allow for the preferential activation of high‐value sequences. One could argue that value (or reward)‐dependent neural activity in CA1 is merely one aspect of CA1’s event‐related neural activity. Although we do not argue against this possibility, we propose that the selection of high‐value sequences is a consequence of CA1’s value‐dependent neuronal activity and that this aspect may represent a core function of CA1. Note that we do not argue that CA1 is solely responsible for value‐dependent processing of replayed sequences. Offline reactivations of specific neural activity patterns that are coordinated with hippocampal replays have been found in value‐related brain structures such as the ventral striatum and VTA (Gomperts, Kloosterman, & Wilson, 2015; Ji & Wilson, 2007; Lansink, Goltstein, Lankelma, McNaughton, & Pennartz, 2009). We propose that the hippocampus, as one of the central components required for imagination and mental simulation, generates novel sequences (CA3) and also filters/reinforces high‐value sequences (CA1). Such filtered sequences may be further associated with value‐related neural activity elsewhere (such as the ventral striatum and VTA) for the control of various psychological processes. These may include the modulation of long‐term storage of CA1 output sequences and the control of motivation for different action sequences. All of this remains to be studied.

Our model concerns reward/value‐dependent CA3–CA1 neural processes. However, the hippocampus contributes to reward‐independent learning as well. For example, hippocampal damages impair simple association learning (such as associating face and name) in humans (Giovanello, Verfaellie, & Keane, 2003; Hannula, Tranel, & Cohen, 2006; Konkel, Warren, Duff, Tranel, & Cohen, 2008; Kroll, Knight, Metcalfe, Wolf, & Tulving, 1996; Stark, Bayley, & Squire, 2002; Turriziani, Fadda, Caltagirone, & Carlesimo, 2004). Also, the hippocampus represents spatial layout of an external environment (cognitive map) even in the absence of reward (Lee, Kim, Sun, & Jung, 2009; McHugh, Blum, Tsien, Tonegawa, & Wilson, 1996; O'Keefe & Nadel, 1978; O'Neill et al., 2008; Spiers, Hayman, Jovalekic, Marozzi, & Jeffery, 2015), which can explain latent learning (Blodgett, 1929; Spence & Lippitt, 1946; Tolman & Honzik, 1930). How is our model related to reward‐independent learning by the hippocampus? First of all, our model does not preclude other types of learning than reward‐based learning in CA3‐CA1 neural network. What we propose is that CA3–CA1 synaptic plasticity/learning is *modulated* by value so that sequential discharges of CA1 neurons are more biased toward reward locations. Second, from the standpoint of hippocampal neurons, “reward” may not be limited to the primary reward. Let's assume, for the sake of argument, that dopamine controls value‐dependent hippocampal neural processes. Dopaminergic neuronal activity is regulated not only by a primary reward, but also by other factors such as novelty (Bromberg‐Martin et al., 2010; Lak, Stauffer, & Schultz, 2016; Ljungberg, Apicella, & Schultz, 1992; Schultz, 1998) and motivation (Bromberg‐Martin et al., 2010; Matsumoto & Hikosaka, 2009; Satoh, Nakai, Sato, & Kimura, 2003). The influence of dopaminergic neurons on the hippocampus may be similar across different occasions such as encountering an unexpected primary reward, exploring a novel environment (e.g., in latent learning), and being motivated for good performance (e.g., in a simple association learning task). This possibility is supported by the findings that novelty lowers the threshold for CA1 long‐term potentiation in a dopamine‐dependent manner (Li et al., 2003) and enhances reactivation of waking CA1 neuronal activity patterns during SWRs (Cheng & Frank, 2008; Foster & Wilson, 2006; O'Neill et al., 2008). Third, other subregions of the hippocampus may be in charge of reward‐independent learning. For example, the DG may bind different types of incoming sensory information (e.g., spatial and nonspatial information) to represent distinct spatial contexts for different environments (Kesner, 2007; Lee & Jung, 2017). The DG may play a major role in simple association learning and latent learning based on its role in “binding”, whereas CA3 and CA1 may primarily concern reinforcing potential valuable sequences.

## 7 PREDICTIONS

Our model allows several predictions. First, both CA3 and CA1 may show replays for novel trajectories during SWRs. Second, the content of SWR‐associated replays may be only weakly modulated by value in CA3, but strongly modulated in CA1 for both experienced and novel trajectories. There is limited evidence for this prediction. Trajectories reconstructed from replays of CA1 place cells are preferentially directed to reward locations (Foster & Wilson, 2006; Gupta et al., 2010; Olafsdottir et al., 2015; Pfeiffer & Foster, 2013; Singer & Frank, 2009), but additional studies are needed. Third, unlike in CA1 (Wikenheiser & Redish, 2013), forward and reverse replays may be equally frequent in CA3 during post‐run sleep. Fourth, if CA3–CA1 synaptic plasticity is blocked during exploration, the value dependence of CA1 replays should be reduced. Fifth, the activity of CA1 place cells during exploration should depend on the expected value associated with their place fields (i.e., the distance to a reward location), but this relationship should be weaker for CA3 place cells (c.f., Lee et al., 2017). Sixth, blocking hippocampal synaptic plasticity during post‐run sleep should reduce novel trajectory‐related hippocampal activity during subsequent navigation. Seventh, assuming value‐dependent CA1 place cell activity during exploration shapes CA3–CA1 synaptic strength (Figure 3), its manipulation should affect value‐dependent replays of CA1 neurons during SWRs. For example, if dopamine plays a role in value‐dependent place cell activity during exploration, then dopamine stimulation (or blockade) during exploration should enhance (or diminish) value‐dependent replays of CA1 neurons during SWRs (c.f., McNamara et al., 2014). Alternatively, if the combination of reverse replays and phasic dopamine release shapes CA3–CA1 synaptic strengths according to value (Figure 5b,c), then dopamine manipulation during reverse replays at a reward location should alter value‐dependent replays of CA1 neurons during SWRs.

## 8 CONCLUSION

Here, we proposed a new model of hippocampal function—the simulation‐selection model—to account for circuit‐level operations of hippocampal information processing for goal‐directed behavior. According to the model, CA3 generates both experienced (remembered) and unexperienced (imagined) firing sequences, while CA1 preferentially passes on and reinforces high‐value sequences. We propose that CA1’s role in selection is made possible by value‐dependent changes in CA3–CA1 synaptic strength, and there is limited evidence that dopamine plays a role in this process. We argue that the simulation‐selection organization of the hippocampus has evolved in mammals, but not in birds, because of the unique ecological and navigational needs of land animals. Although solid empirical evidence is missing for many aspects of our model, we hope to revise this model as empirical results accumulate. In particular, it is critical to understand how CA3 and CA1 neural activity is modulated by value during different behavioral states. We have also only considered CA3 and CA1 as central components of the simulation‐selection model, leaving out the DG, CA2, and the subiculum. Our model will require correction and expansion as these subfields are incorporated. Nevertheless, we hope our model provides a new perspective for the field of hippocampal mnemonic processing as well as testable hypotheses that can guide future empirical work on the hippocampus.

## CONFLICT OF INTEREST

The authors declare no competing interests.
